# Accelerating TB regimen development: introducing FAST-TB

**DOI:** 10.5588/ijtldopen.24.0333

**Published:** 2024-11-01

**Authors:** T. Devezin, C. Chisholm, F. Jones, K. Lacourciere, B. Laughon, L. Ramachandra, A. Vernon, L. Zhang, P. Kim, C. Lienhardt

**Affiliations:** ^1^ CRDF Global, Arlington, VA, USA;; ^2^ Division of AIDS, National Institute of Allergy and Infectious Diseases, National Institutes of Health, Bethesda, MD, USA;; ^3^ Division of Microbiology and Infectious Diseases, National Institute of Allergy and Infectious Diseases, National Institutes of Health, Bethesda, MD, USA.

**Keywords:** tuberculosis, therapeutics, drugs, drug development

After decades of stagnation, research into new treatments for TB is experiencing a renaissance, with an increasing number of new and repurposed drugs being evaluated in novel treatment regimens.^1^ For example, the WHO now recommends a new 4-month regimen for the treatment of drug-susceptible TB,^2,3^ and new fully oral treatments of 6-month duration are recommended for the treatment of drug-resistant TB.^4^ With several new medicines expected to enter clinical testing within the next five years, the possibility of safer, shorter and more effective treatment regimens appears in reach.^5^ Recent clinical studies have provided valuable insights for future clinical development,^6^ and it is anticipated that trials evaluating the next generation of drugs and regimens will lead to more effective and safer treatment options, which are desperately needed.^7^ However, even with these recent advances, clinical development of new TB therapeutics faces the challenges of identifying the best combination of drugs offering optimal efficacy and safety, along with the optimum dose of each drug and the optimal treatment duration to achieve a relapse-free cure,^7,8^ In addition, clinical testing should proceed with as little risk as possible. These long and costly Phase III trials would greatly benefit from the availability of reliable markers of sustained therapeutic efficacy, together with agile modeling tools to predict the quantitative relationship between Phase II and Phase III clinical trial outcomes.^9,10^ The WHO recently updated the target regimen profiles for TB treatment, aimed at guiding drug developers to produce regimens that are quality-assured, affordable, widely available and meet the needs of affected populations.^11^ Although several clinical trial networks and consortia are independently advancing research to test promising regimens, there is no platform to facilitate communication and collaboration between them. Such a platform could accelerate and streamline the development of new treatment regimens by establishing clear and rational approaches for the identification of suitable drug combinations, including novel trial designs with appropriate endpoints and analytic strategies, as well as harmonized data collection.^7,10,12^ Ideally, these should be complemented by early knowledge of the drugs’ pharmacokinetic (PK), pharmacodynamic (PD), and bactericidal characteristics, together with detailed information on the drug’s effects in patient populations.^13,14^

**Figure 1.**
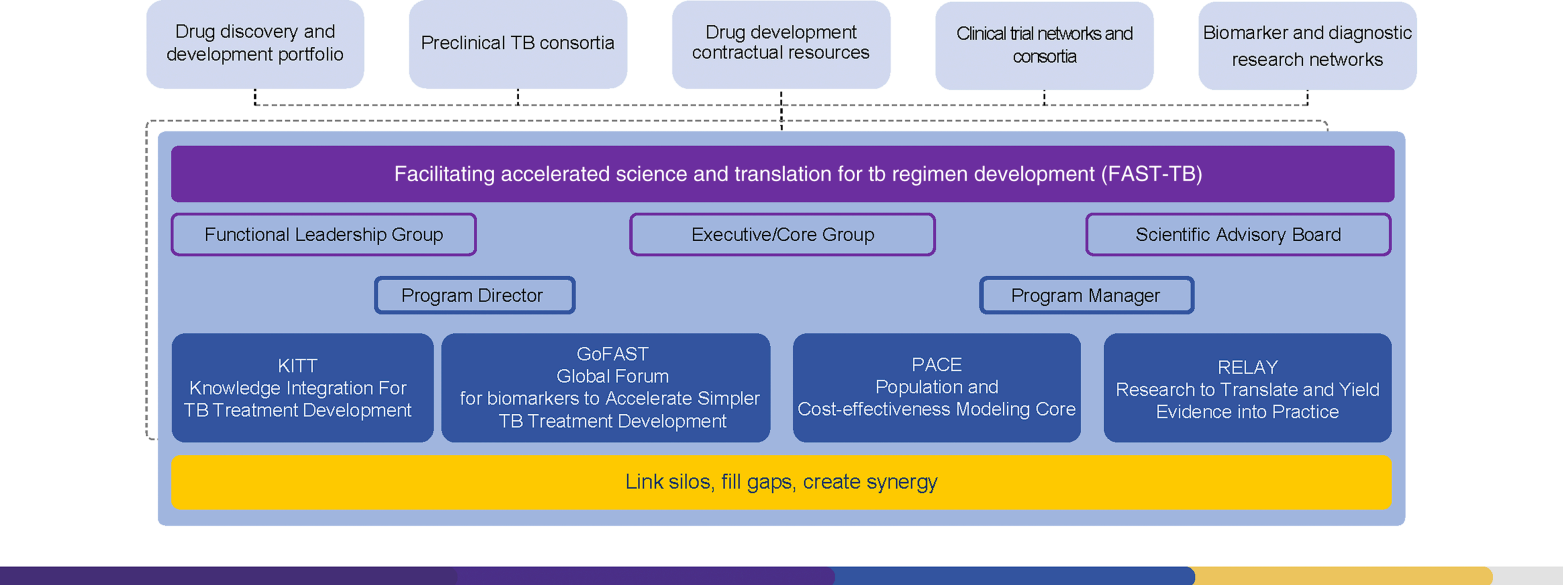
FAST-TB leadership structure. FAST-TB = Facilitating accelerated science and translation for TB regimen development.

The Global Plan to Stop TB estimated that USD6.8 billion would be required to support TB drug research during 2018–2022. However, global investment in TB drug development has fallen short of this target by over USD5 billion.^15^ Given these challenges and the limited funding allocated for TB research, it is crucial to streamline best practices for the development of new treatments while addressing gaps and avoiding duplication of effort. In response to this need, the US National Institute of Health, National Institute of Allergy and Infectious Diseases (NIH/NIAID), in collaboration with CRDF Global, has established the Facilitating Accelerated Science and Translation for TB Regimen Development (FAST-TB) program. This is a platform to coordinate more efficient, harmonized and streamlined processes for developing novel TB treatment regimens and their subsequent delivery to those in need in high TB burden settings. Through its focus on promoting better communication, coordination and collaboration among key stakeholders and research groups along the TB drug development pathway, FAST-TB has the following primary objectives: support sharing of preclinical/clinical knowledge and data to accelerate the progress of suitable regimens along the clinical development pathway; support and facilitate research to develop and validate biomarkers to advance the development of new TB treatments; support and facilitate research to better understand factors associated with drug resistance and development of easily implementable assays for drug susceptibility testing (DST) during drug development; facilitate a research strategy to optimize the introduction of new TB regimens in high burden countries, one that is informed by a nuanced understanding of real-world needs and fosters engagement with relevant stakeholders. To support the implementation of these objectives, FAST-TB operates the following four Tracks (Figure 1): 1) Knowledge Integration for TB Treatment development (KITT); 2) Global Forum for Biomarkers to Accelerate Simpler TB Treatment (GoFAST); 3) Population and Cost-Effectiveness Modeling Core (PACE); and 4) Research to Translate and Yield Evidence for Practice (RELAY).

The first track (KITT) aims to establish a collaborative framework for stakeholders involved in TB drug development to share preclinical and clinical knowledge, plans and ideas to facilitate and reinforce the clinical development of novel TB treatment regimens. KITT integrates novel developments in PK/PD, microbiology and pharmacometrics,^9,13^ as well as in the design of clinical trials.^14^ KITT is fostering a worldwide community comprising consortia, networks, and groups engaged in the development of TB treatments. This community will exchange information about trial plans, designs, and experimental data on a global scale. Through early sharing of preclinical and clinical knowledge, KITT aims to stimulate the creation of model-informed drug development frameworks for a new generation of knowledge-driven TB regimens. GoFAST aims to establish a global forum of TB researchers and stakeholders to promote the advancement and validation of novel biomarkers, diagnostics, and drug resistance assays and inform the clinical development and use of new TB drugs and regimens.^16^ Biomarkers that can quantitatively assess the sterilizing capacity of new drugs and drug combinations and predict long-term treatment effects more reliably and faster than current culture-based methods may help to speed up their progression along the treatment development pathway.^17,18^ In addition, following new recommendations from the updated WHO target regimen profiles,^11^ GoFAST aims to promote the development of DST assays for new TB drugs in clinical development. Parallel development of DST assays will provide data for early decision-making to advance new drugs and regimens in clinical trials. The identification, validation and integration of drug resistance markers into existing diagnostic platforms will enable prompt adjustments to regimens to improve treatment outcomes and prevent the emergence of further drug resistance. Such biomarkers should be easy to interpret and portable across clinical phases and provide the necessary data to inform rank ordering and prioritization of regimens for clinical evaluation. The PACE Modeling Core will address and rapidly respond to FAST-TB’s modeling needs by estimating the impact and cost-effectiveness of novel treatment regimens and diagnostic strategies. PACE will consider the background epidemiological features of populations and specific characteristics of new treatment regimens to inform which research priorities and product profiles have the greatest public health impact, including among vulnerable populations. It is expected that FAST-TB partners and stakeholders will use the generated data and estimates to support evidence-based decision-making. Findings will be disseminated to enable all stakeholders to take rapid action at the global and national levels. Finally, to ensure that clinical research strategies for treatment development are informed by a nuanced understanding of real-world needs,^19^ RELAY intends to connect all relevant parties. This includes national TB Programs, TB-affected communities, regulatory and normative bodies, as well as other groups responsible for introducing new TB regimens into care and practice and with research groups working upstream. This bottom-up loop will ensure that research efforts are informed by the needs and priorities of TB-affected communities and the wide range of inputs from health workers involved in TB care. RELAY will focus on the global realities of TB care and offer an opportunity for TB-affected communities and health workers to influence upstream research efforts.

**Figure 2.**
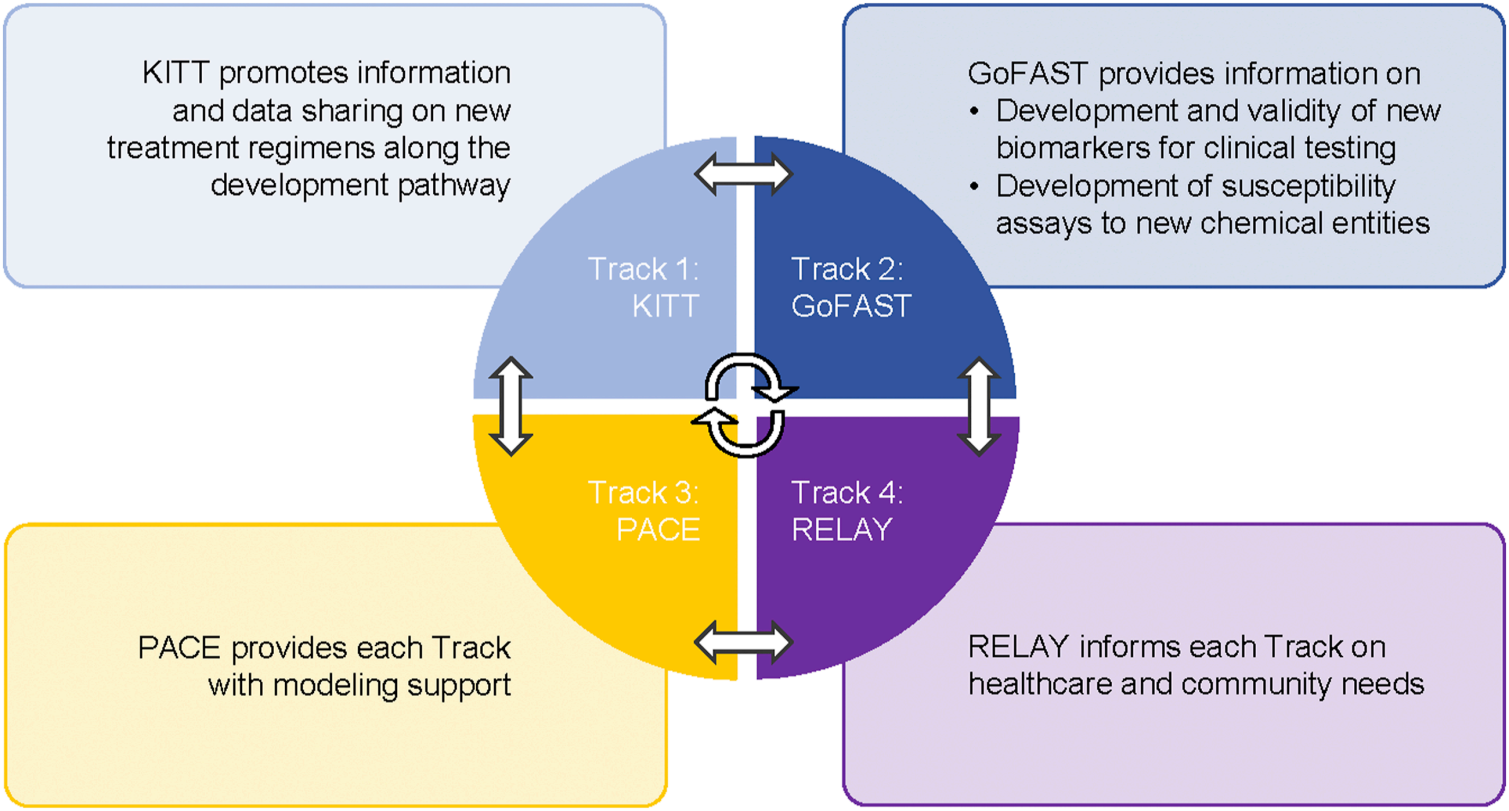
FAST-TB track communication flow. KITT = Knowledge Integration for TB Treatment development; GoFAST = Global Forum for biomarkers to Accelerate TB Treatment Development; PACE = Population and Cost-effectiveness Modeling Core; RELAY = Research to Translate and Yield Evidence into Practice; FAST-TB = Facilitating accelerated science and translation for TB regimen development.

To ensure the most dynamic approach to address the identified gaps and limitations in TB treatment research, these four FAST-TB Tracks will work synergistically along the drug development pathway with regular exchanges, meetings and the creation of reference papers (Figure 2). By identifying gaps along the treatment development pathway and using creative approaches in translational science to bridge existing silos, FAST-TB will contribute to improving the systematization and harmonization of best practices and research designs to accelerate the development of new TB regimens.^20^ These will include fostering preclinical and clinical collaborative development, conducting scientific workshops, providing expert input in TB modelling strategies and promoting stakeholder and community engagement. By establishing a collaborative environment for organizations, agencies and institutions working at different stages of the treatment development pathway – and creating synergies among a wide range of stakeholders – FAST-TB will support a more efficient and streamlined process for developing and evaluating novel treatments. Ultimately, the goal of FAST-TB is to speed up research to benefit everyone affected by TB worldwide.
